# Multilevel Cervical Epidural Hematoma From C1 to T3 Following C6/C7 Anterior Cervical Discectomy and Fusion (ACDF): A Rare but Reversible Surgical Emergency

**DOI:** 10.7759/cureus.87295

**Published:** 2025-07-04

**Authors:** Evangelos Christodoulou, Alexandros Christodoulou

**Affiliations:** 1 Orthopedics and Trauma Surgery, Clinic for Spine and Pain, St. Vinzenz Hospital, Düsseldorf, DEU; 2 Orthopedics and Trauma Surgery, Helios St. Johannes Klinik, Duisburg, DEU

**Keywords:** acdf complications, catheter, hydrogen peroxide, minimally invasive, multilevel cervical epidural hematoma, postoperative complication, postoperative spinal epidural hematoma (seh), revision surgery, spinal decompression, tetraparesis

## Abstract

Postoperative spinal epidural hematoma (SEH) is a rare but potentially devastating complication following anterior cervical discectomy and fusion (ACDF). We report a case of a 60-year-old man who developed an extensive cervical SEH from C1 to T3 after undergoing C6/C7 ACDF. The patient presented with acute tetraparesis and respiratory failure necessitating immediate intubation. A computed tomography (CT) scan revealed a multilevel epidural hematoma extending anteriorly from the craniovertebral junction to the upper thoracic spine. We performed an urgent anterior revision surgery. The previously inserted ACDF cage was removed, and two ventricular catheters (Neuromedex GmbH, Hamburg, Germany), with an outer diameter of 3.0 mm and an inner diameter of 1.5 mm, were inserted cranially and caudally for saline lavage. Active bleeding was identified posterior to the C7 vertebra, which did not respond to conventional hemostatic sponges but was successfully controlled using hydrogen peroxide (H_2_O_2_). Postoperatively, the patient exhibited immediate neurological recovery and was discharged without deficits on postoperative day five. Follow-up CT imaging demonstrated complete resolution of the hematoma. This case emphasizes the need for high suspicion and prompt surgical management in cases of postoperative SEH. Our technique may offer a valuable minimally invasive method for hematoma evacuation in similar emergencies.

## Introduction

Cervical spinal epidural hematoma (SEH) is an uncommon but potentially catastrophic postoperative complication, particularly following anterior cervical spine surgery such as anterior cervical discectomy and fusion (ACDF). The incidence is reported to be less than 1% [1], yet when it occurs, the consequences can include acute spinal cord compression, neurological deterioration, and in severe cases, respiratory failure and death [2,3]. Early recognition and timely intervention are critical to preventing permanent neurological deficits. While revision through the primary anterior access is preferred when the SEH is located at the index level, there is no consensus on the optimal strategy in rare cases involving extensive hematomas spanning multiple levels. Management options include posterior multilevel laminectomies and anterior single- or multilevel decompression, each associated with significant risks and technical challenges [4-8].

In this report, we describe a rare case of a multilevel SEH extending from C1 to T3 in a 60-year-old male following C6/C7 ACDF. The hematoma resulted in tetraparesis and respiratory compromise. We detail a successful anterior revision approach utilizing ventricular catheter lavage and hydrogen peroxide (H_2_O_2_)-assisted hemostasis, leading to full neurological recovery.

## Case presentation

**Figure 1:**
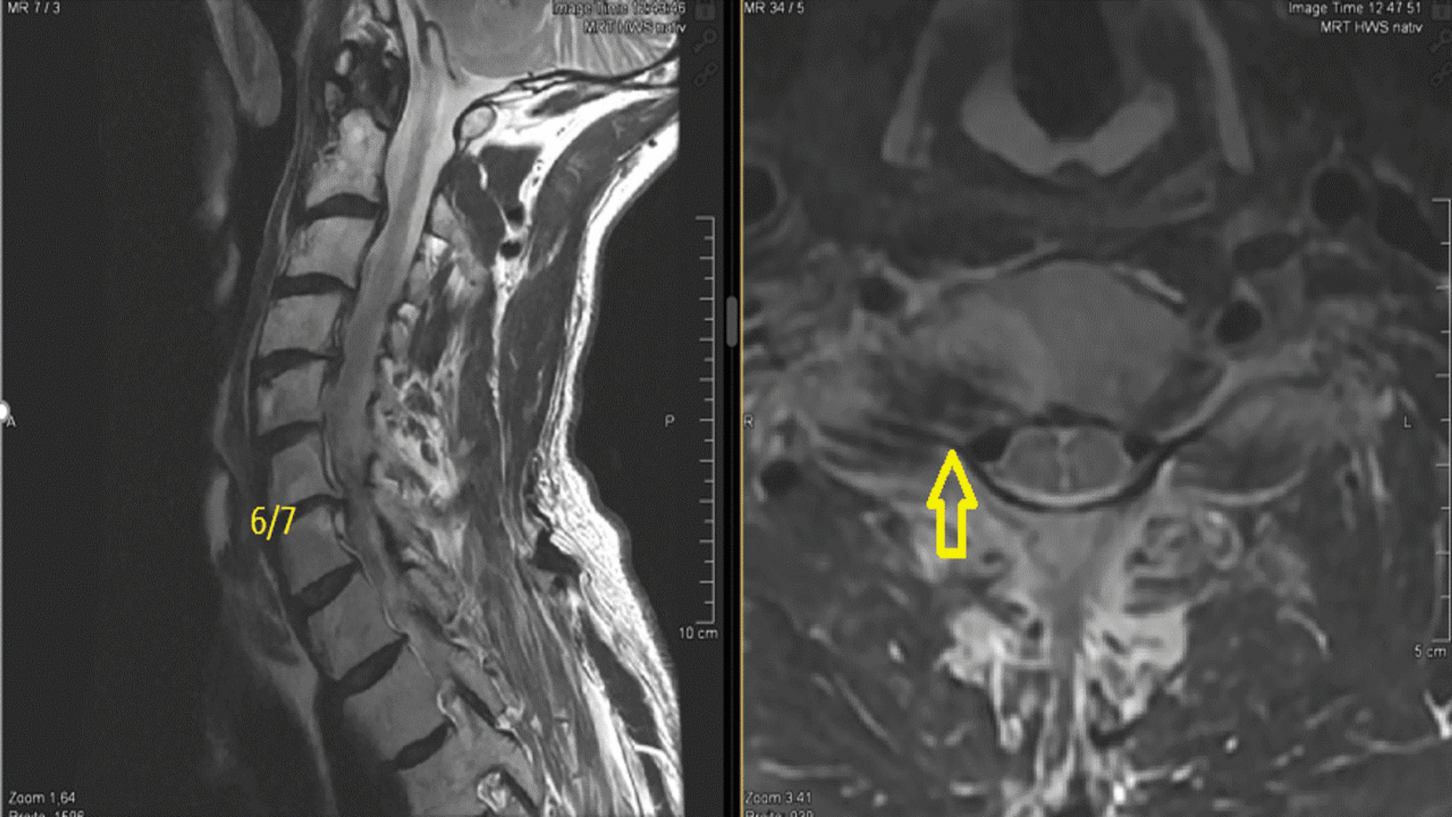
Preoperative T2-weighted MRI (sagittal and axial views) showing right-sided neural foraminal stenosis at the C6-C7 level (yellow arrow). MRI, magnetic resonance imaging

A 60-year-old man was admitted to our hospital with cervical and right arm pain consistent with C7 radiculopathy. On neurological examination at admission, he exhibited no motor deficits but had persistent paresthesia in the C7 dermatome. Magnetic resonance imaging (MRI) revealed degeneration of the C6-C7 intervertebral disc, along with neuroforaminal stenosis at the same level (Figure 1).

His medical history included hypercholesterolemia and arterial hypertension, for which he was receiving regular medication. He had also been on prophylactic aspirin (100 mg daily), which was paused upon admission and discontinued entirely 10 days prior to the planned surgical intervention.

After an unsuccessful attempt at conservative management, including a computed tomography (CT)-guided C7 nerve root block, the patient underwent a standard ACDF, as recommended in current literature [9]. The surgery was uneventful, with an operative time of 71 minutes and an estimated intraoperative blood loss of 150 mL. The initial postoperative neurological examination following extubation revealed no deficits.

**Figure 2:**
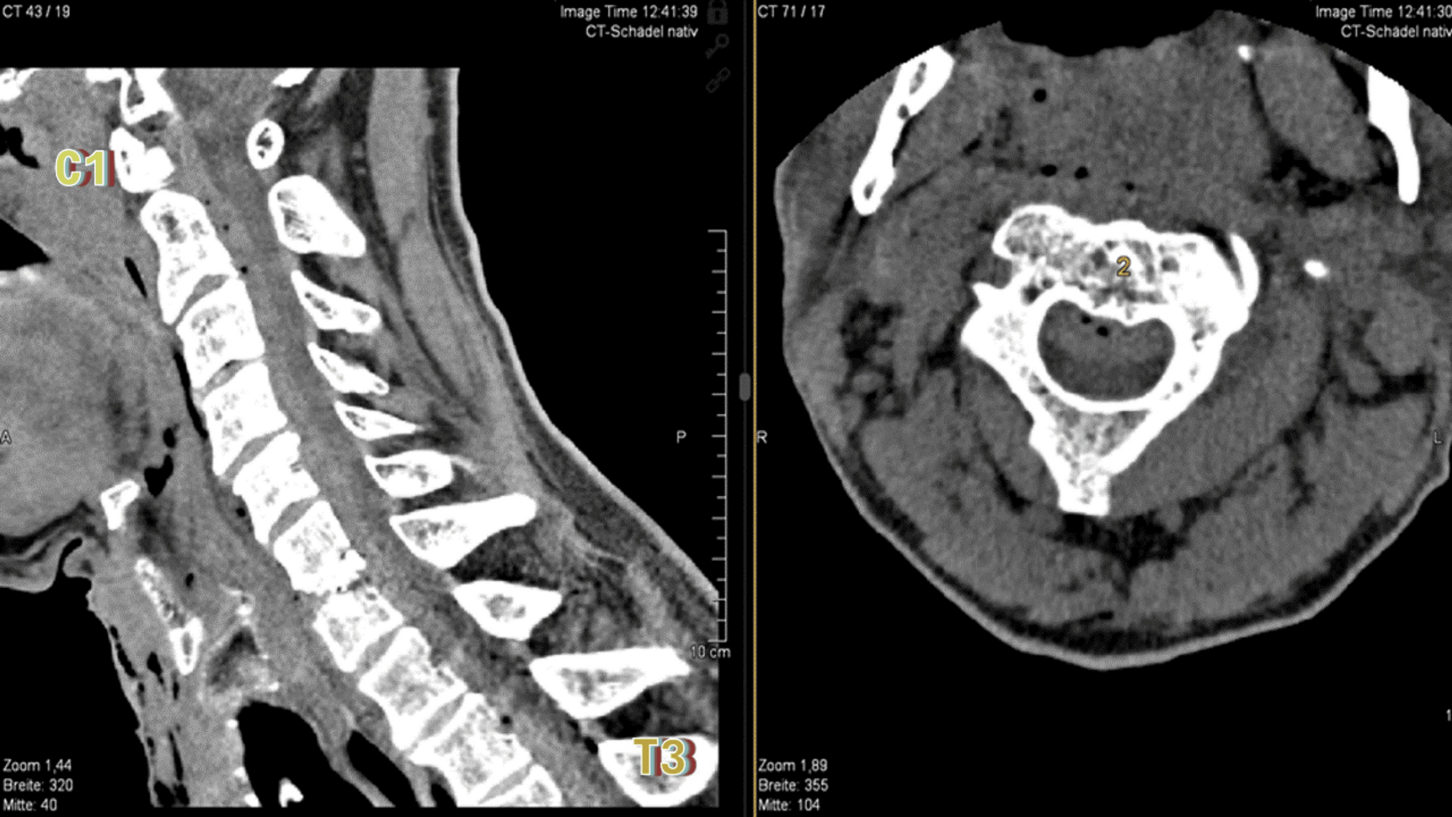
Postoperative CT imaging (sagittal and axial views) demonstrates a multilevel epidural hematoma extending from C1 to T3. CT, computed tomography

However, approximately two hours postoperatively, the patient reported difficulty breathing and progressive muscle weakness, which rapidly advanced to tetraparesis and ultimately complete tetraplegia. He required emergency endotracheal intubation. An urgent CT scan revealed an extensive intraspinal epidural hematoma extending from C1 to T3, with an estimated volume exceeding 20 cm³ (Figure 2). The indication for urgent revision surgery was immediately established. The time interval between the onset of symptoms and the initiation of revision was approximately three hours.

**Figure 3:**
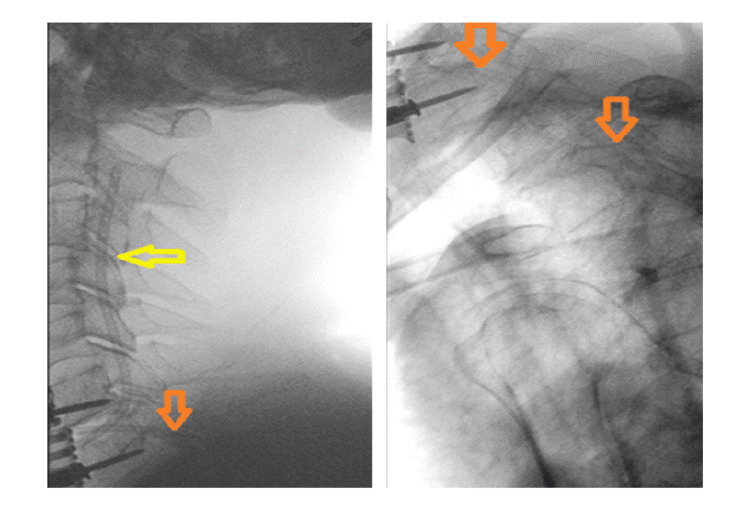
Intraoperative placement of two ventricular catheters into the epidural space: one cranially (yellow arrow) and one caudally (orange arrows), allowing continuous irrigation with normal saline.

Intraoperatively, the previously placed ACDF cage was removed to facilitate decompression. Two ventricular catheters (Neuromedex GmbH, Hamburg, Germany; 3.0 mm outer diameter, 1.5 mm inner diameter) were inserted into the epidural space, one cranially and one caudally, enabling continuous irrigation. Approximately 500 mL of normal saline was used over a period of 30 minutes to flush out residual hematoma and promote spinal cord decompression (Figure 3). 

A bleeding source was identified posterior to the C7 vertebra. Standard hemostatic agents, including absorbable gelatin sponge and a gelatin matrix combined with thrombin, were applied but failed to achieve adequate hemostasis. Hemostasis was ultimately achieved using approximately 30 mL of 3% H₂O₂, which effectively controlled the bleeding. The use of H₂O₂ was uneventful, with no observed local or systemic complications.

**Figure 4:**
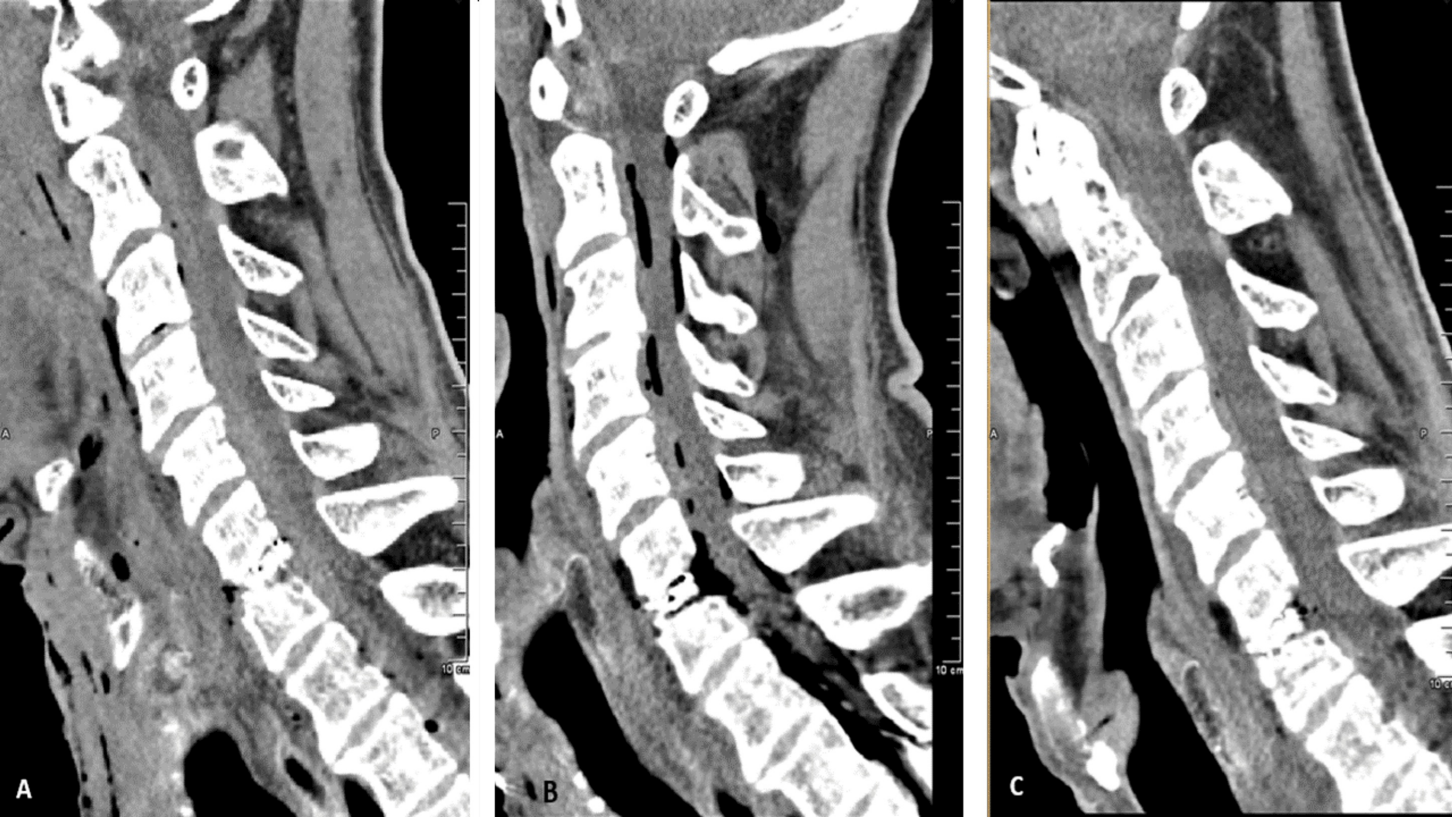
Sagittal CT imaging during the course of treatment: (A) Postoperative scan following ACDF showing an extensive epidural hematoma extending from C1 to T3. (B) Immediate postoperative scan after revision surgery demonstrating a small residual hematoma and postoperative air. (C) Follow-up scan five days after revision surgery confirming complete resolution of the hematoma. ACDF, anterior cervical discectomy and fusion

The patient demonstrated immediate and total neurological recovery postoperatively. He was extubated on the same day to assess neurological recovery and discharged home on day 5 without neurological deficits. Follow-up CT imaging confirmed complete resolution of the hematoma (Figure 4).

## Discussion

SEH following ACDF is a rare (0.4% to 1.2%) but serious complication that necessitates early recognition and timely surgical intervention. Several risk factors have been associated with SEH, including diffuse idiopathic skeletal hyperostosis (DISH), ossification of the posterior longitudinal ligament (OPLL), multilevel surgery, smoking, alcohol consumption (>10 units per week), and hypertension [10,11]. There are cases where hematoma formation may occur postoperatively despite careful intraoperative hemostasis. Contributing factors can include coagulation abnormalities, surges in blood pressure during anesthesia emergence, or increased venous pressure caused by coughing or straining, such as during extubation and the associated Valsalva maneuver [12].

In most cases, SEH occurs at the index level and primary surgical site. Multilevel SEH following ACDF has been reported only a few times in the literature (Table 1) [4-8,13,14]. Symptoms may range from severe neck and arm pain, cyanosis, and dyspnea to progressive paraplegia or tetraparesis [7,8,13]. In our case, the patient developed both tetraplegia and respiratory insufficiency.

**Table 1:** Comparison of previously reported multilevel SEH cases. SEH, spinal epidural hematoma; ACDF, anterior cervical discectomy and fusion

Reference	Levels Involved	Treatment	Use of Drain	Complications	Outcome
Kim JS, Kuh SU (2012) [4]	C2-T5	ACDF revision and blood evacuation	Hemovac tube	None reported	Full recovery at six-month follow-up
Hans P et al. (2003) [5]	C3-T3	Posterior cervical laminectomy and surgical evacuation of the hematoma	No	None reported	Full recovery
Lee DY, Lee SH (2010) [6]	C1-T4	ACDF revision and blood evacuation	Yes	None reported	Immediate recovery
González-Diaz R et al. (2016) [7]	C3-T6	Corpectomy with bladder catheter irrigation	Yes	None reported	Full recovery at one-year follow-up
Cetintas SC et al. (2023) [8]	C1-C6	Multiple posterior laminectomies	Two submuscular drains	Diffuse hypoxic encephalopathy	Exitus letalis
Jang JW et al. (2010) [13]	C1-C6	Conservative management (spontaneous resolution)	No	None reported	Full recovery at two weeks postoperatively
Morace R et al. (2021) [14]	C2-T1	ACDF revision and open-tip lumbar external drainage catheter irrigation	Yes	None reported	Immediate recovery

When neurological symptoms persist, there is no consensus regarding the optimal treatment approach for this potentially devastating complication. In two reported cases, hematoma evacuation was initially performed successfully through the original anterior approach. Posterior laminectomies were considered as a secondary option in case of inadequate neurological recovery [4,6].

Jang et al. (2010) reported a case of multilevel SEH with spontaneous resolution of tetraparesis, in which revision surgery was not required [13].

Lawton et al. (1995) reported that in their series, re-exploration of the original surgical site was performed in all cases. He emphasized that patients taken to surgery within 12 hours had significantly better neurological outcomes [15]. Amiri et al. (2013) observed a median improvement of two Frankel grades in patients who underwent surgical evacuation within six hours, compared to a one-grade improvement in those treated after six hours [16].

In another case, urgent revision through the anterior approach was performed without preoperative CT or MRI. Due to a lack of neurological improvement, an MRI was later obtained, which revealed a multilevel SEH. The patient then underwent a second revision via posterior laminectomies [5]. Cetintas et al. (2023) chose to directly perform multilevel posterior laminectomies for hematoma evacuation [8]. González-Díaz et al. (2016) opted to convert the ACDF to a corpectomy to allow wider decompression and inserted a urinary catheter for saline irrigation [7]. 

In our case, no corpectomy was necessary. Two ventricular catheters were inserted through the same intervertebral space used in the original ACDF. Morace et al. (2021) reported a similar technique, using a lumbar external drainage catheter for complete removal of a multilevel SEH [14].

The use of ventricular catheters for targeted lavage enabled effective evacuation of the hematoma without requiring multilevel laminectomy or corpectomy. Additionally, H₂O₂ was employed as a hemostatic agent when conventional methods failed. Although H₂O₂ has been shown to reduce intraoperative blood loss in spinal surgery, its use must be carefully considered due to rare but serious complications such as gas embolism [17-19]. In our case, the severity of the patient’s condition, tetraplegia and respiratory insufficiency, justified its use after other measures had failed.

While posterior decompression is more commonly employed for multilevel cervical stenosis, it is more invasive if the initial surgery was a one-level ACDF and may not be ideal in all clinical scenarios. In our case, the anterior approach provided direct access through the existing surgical corridor, reducing operative time and tissue disruption.

This technique offers several potential advantages: minimal invasiveness, avoidance of posterior instrumentation, preservation of posterior elements, and reduced surgical morbidity.

This case underscores the importance of rapid diagnosis using imaging, maintaining a low threshold for revision surgery, and timely surgical intervention. It also highlights the value of intraoperative innovation in managing life-threatening complications. The favorable neurological outcome and short hospital stay illustrate the effectiveness of the chosen approach.

## Conclusions

Multilevel cervical SEH is a rare but potentially life-threatening complication following ACDF that requires rapid clinical recognition and immediate action. Our case illustrates how early detection, swift surgical decision-making, and innovation in intraoperative techniques, such as the use of ventricular catheters for lavage and H₂O₂ for hemostasis, can lead to a full neurological recovery even in severe presentations. The anterior approach provided a direct and effective corridor for hematoma evacuation, minimizing further surgical morbidity. This report not only reinforces the importance of vigilant postoperative monitoring but also introduces a reproducible and minimally invasive technique that could aid in managing similar high-risk complications. Further studies are warranted to evaluate the generalizability and safety of this approach in a broader patient population. Effective communication, teamwork, and preparedness were critical in achieving the successful outcome for our patient, and these principles remain essential in the management of complex spinal emergencies.
